# Association between carbohydrate intake and fatty acids in the de novo lipogenic pathway in serum phospholipids and adipose tissue in a population of Swedish men

**DOI:** 10.1007/s00394-019-02058-6

**Published:** 2019-07-26

**Authors:** Zayed D. Alsharari, Karin Leander, Per Sjögren, Axel Carlsson, Tommy Cederholm, Ulf de Faire, Mai-Lis Hellenius, Matti Marklund, Ulf Risérus

**Affiliations:** 1 Department of Public Health and Caring Sciences, Clinical Nutrition and Metabolism, Uppsala University, Box 564, 75122 Uppsala, Sweden; 2 Unit of Cardiovascular Epidemiology, Institute of Environmental Medicine, Karolinska Institutet, Stockholm, Sweden; 3 Division of Family Medicine and Primary Care, Department of Neurobiology, Care Sciences and Society, Karolinska Institutet, Stockholm, Sweden; 4 Cardiology Unit, Department of Medicine, Karolinska University Hospital, Karolinska Institutet, Stockholm, Sweden

**Keywords:** Carbohydrate intake, Fatty acid composition, Saturated fatty acids, De novo lipogenesis, Stearoyl-Co A desaturase (SCD), Alcohol intake

## Abstract

**Purpose:**

Fatty acid composition in blood and adipose tissue (AT) is a useful biomarker of dietary fat quality. However, circulating saturated fatty acids (SFA) and monounsaturated fatty acids (MUFA) have been proposed to also reflect carbohydrate-induced de novo lipogenesis (DNL) and stearoyl-CoA desaturase (SCD) activity. We aimed to test the hypothesis that high carbohydrate intake is related to SFA and MUFA in serum or AT in a Swedish population.

**Methods:**

Fatty acid composition was measured in serum phospholipids (PL) and AT by gas chromatography in 63-year-old men (*n* = 299). Carbohydrate and alcohol intake was assessed (validated 7-day food records) in relation to total SFA, 16:0 (palmitate), 16:1 (palmitoleate), and estimated SCD activity (16:1n-7/16:0-ratio) in serum PL and in AT, respectively.

**Results:**

Total carbohydrate intake was inversely associated with 16:0 in PL (*P* = 0.005), independently of BMI. Disaccharides were non-linearly (restricted cubic splines) and weakly associated with 16:1 and SCD activity in PL (nonlinear trend, *P* ≤ 0.02) but not AT. Carbohydrate intake and SCD expression were not associated (*P* ≥ 0.08, *n* = 81). Alcohol intake was, however, linearly associated with 16:0 in PL (*P* < 0.001), and with 16:1 (*P* < 0.001) and SCD activity (*P* ≤ 0.005) in both PL and AT.

**Conclusions:**

Higher carbohydrate intake from sugar-rich foods or beverages was not clearly reflected by higher SFA or SCD activity in serum PL or AT. Alcohol was, however, associated with higher SFA and MUFA.

**Electronic supplementary material:**

The online version of this article (10.1007/s00394-019-02058-6) contains supplementary material, which is available to authorized users.

## Introduction

Fatty acid (FA) composition in plasma lipids and adipose tissue (AT) are widely used as biomarkers of dietary fat quality. However, whereas essential polyunsaturated FAs are overall useful biomarkers of PUFA intake, even-chain saturated fatty acids (SFA) and monounsaturated fatty acids (MUFA) are weaker biomarkers of dietary intake. This discrepancy among FA biomarkers may in part be due to the fact that SFA are non-essential and are thus to some extent endogenously synthesized. In addition, the major SFA palmitic acid (16:0) seems to be tightly regulated and can be readily desaturated to MUFA by stearoyl-CoA desaturase (SCD) [1].

De novo lipogenesis (DNL) is a process of converting excess carbohydrate (and alcohol) into even-chain SFA and subsequently to MUFA [2]. 16:0 is the major dietary SFA, but may also be endogenously synthesized through DNL during certain dietary conditions. Interventional studies suggest that hepatic DNL is induced by a high-carbohydrate and low-fat diet [3], leading to increased proportions of circulating 16:0 and other even-chain SFA [4–6]. Further, overfeeding studies suggest that hepatic DNL is increased in healthy subjects overfed with carbohydrates [7]. Observational data are scarce, but in a US population of elderly individuals, there was a positive association between higher carbohydrate intake replacing fat and alcohol, and plasma SFA in phospholipids (PL) [8–10]. In the EPIC-interact study, there were inconsistent associations between carbohydrate-rich foods and SFA in plasma PL, but still a possible role of DNL in explaining the increased risk of even-chained SFA with type 2 diabetes [11].

To our knowledge, no studies have investigated carbohydrate intake and the association with both circulating and AT SFA, SCD activity and *SCD* gene expression in the same population. Since the AT SFA turnover is lower than in serum lipids, and also that DNL may occur in AT, we found it of high interest to investigate FAs also in AT. Yet no studies have been conducted in Scandinavian populations, despite that several Swedish prospective cohort studies have demonstrated a direct link between serum SFA (especially 16:0) and diabetes and CVD [12, 13]. To interpret these associations between serum SFA and increased risk of disease outcomes, dietary and food intake data of carbohydrates and sugars are of clear interest. The aims of this study were to evaluate the association between carbohydrate, sugar, and alcohol intakes with individual SFA in the DNL pathway and total even-chain SFAs and MUFA in serum phospholipid and adipose tissue. Also, the associations between carbohydrates, SCD activity and *SCD* gene expression were evaluated. We hypothesized that higher intakes of total carbohydrates or sugar-rich foods, and/or alcohol intakes may be reflected in serum PL and AT as higher proportions of 16:0, 16:1, total SFA and possibly also as increased estimated SCD activity and AT gene expression.

## Methods

### Study population

The study population has been described previously [14]. In brief, a cross-sectional study of 301 healthy men aged 63 years was conducted between March 2000 and October 2001. The men were recruited from a cohort study of 60-year-old men and women (60YO) who had a baseline investigation between 1997 and 1999 regarding risk factors for cardiovascular disease. Participants of 60YO were born in Sweden, had BMI between 20 and 35 kg/m^2^, were without CVD, had no pharmacological treatment of hypertension, diabetes or hypercholesterolemia, and had no other serious disease. Men were divided into three groups based on tertiles of their fasting insulin concentrations. Requests to participate in a study concerning diet and metabolic syndrome were overall randomly sent until positive responders reached approximately 100 men in each group. Classification of groups was used only to recruit subjects with a wide range of insulin concentrations and not for analyses in this study.

### Clinical procedures

The participants underwent a medical examination including fasting blood sampling in the morning and anthropometric measurements. Information was collected about their medications, physical activities and smoking histories during interview. Written and oral instructions were given individually about how to complete 7-day food record. Participants were told not to change their eating habits during the study time and they returned with completed food record and urine samples after approximately 1 week. A biopsy of subcutaneous AT from the left upper buttock was taken using a 1.2 × 50-mm needle after applying an anaesthetic cream (Emla 5% (Astra), containing 2.5% lidocaine and 2.5% prilocaine) on skin for 20–30 min [15].

### Dietary data

The dietary assessment method has been described previously [14, 16]. Briefly, food record was completed during seven consecutive days by an optically readable version of a questionnaire used in a national dietary survey performed in 1989 by the Swedish National Food Administration and Statistics Sweden [17, 18]. Intake of food and nutrients was calculated using the food composition database of the Swedish National Food Administration (PC-Kost, version 1/99; Swedish National Food Administration) and SAS software (SAS Institute Inc, Cary, NC USA) [19]. Individual food items were grouped into main food groups such as milk, bread, and fruits.

Macronutrient intake were calculated based on intake of energy for carbohydrates, fiber, fat, protein, and alcohol. Total energy intake was expressed as megajoules per day (MJ). The intake of food groups was expressed as gram per 10 MJ. Intake of carbohydrates: total carbohydrate, disaccharide, monosaccharide, and fiber was expressed as percentages of total energy intake (%). Also, alcohol intake was expressed as percentages of total energy intake. Intake of fat: total fat, saturated fat, polyunsaturated fat, and monounsaturated was expressed as percentages of total energy intake.

Food groups were created based on the estimated level of carbohydrate contents. Three food groups were defined; the sugar-rich food group included sugar, syrup, honey, candy, chocolate, jam, soft drinks, lemonade, juices, ice cream, desserts, cookies, crackers and buns. The starch-rich food group included bread, cereals, porridge, pancakes, pizza, pasta, potatoes, and rice. The fruit and vegetable food group included fruit, berries, vegetables, and root vegetables.

### Fatty acid composition in serum phospholipids and adipose tissue

Briefly, serum and fat biopsies were stored up to 1 year at − 70 °C until FA measurement by gas–liquid chromatography [20] which was conducted as previously described in detail [21]. SCD activity was estimated as the 16:1/16:0 ratio. Total even-chain SFA was defined as the sum of 14:0, 16:0 and 18:0. The relative amount of FA was expressed as the percentage of the sum of all fatty acids detected.

### SCD gene expression

For gene expression analysis, a subsample of 87 individuals was selected from all men who had fat biopsies collected and were equally distributed throughout the tertiles of fasting insulin concentration. The laboratory procedures of measuring SCD expression have been previously described [22, 23]. Briefly, SCD mRNAs were quantified by real-time polymerase chain reaction and normalized for the expression of the housekeeping gene *RPLP0* [22].

### Statistical methods

Median and interquartile range (IQR) calculated for general characteristics such as clinical measurement and anthropometrics were presented. Circulating FA proportions, SCD activities and gene expression ratios were presented as mean and 95% confidence interval (CI). Spearman rank correlation coefficients and their 95% CI were calculated to evaluate the relationship between carbohydrate intake and FAs, sum of SFA, and SCD. Skewed variables were logarithmically transformed. Associations of nutrient intakes (i.e. carbohydrates, carbohydrate-to-fiber ratio, di- and monosaccharides, and alcohol) with FAs (i.e. 16:0, 16:1, and sum of SFA), SCD activity, *SCD* gene expression, and plasma triglycerides were assessed in linear regression models, with FAs, SCD activity, *SCD* gene expression, or plasma triglycerides as dependent variables and tertile median intake as independent variable. Crude associations were adjusted for BMI. Non-linear trends were assessed using restricted cubic splines with three knots and BMI as a covariate. In a post hoc exploratory analysis, association of disaccharide intake with FAs or SCD activity was assessed (with and without adjustment for BMI) in linear regression models, stratified by disaccharide intake (i.e. < 10%E versus ≥ 10%E), with FAs or SCD activity as dependent variable and disaccharide intake as a continuous independent variable. *P* values < 0.05 were considered significant. Statistical analyses were carried out by STATA version 13.0 (STATA Corporation, TX, USA).

## Results

Of the 301 men, two were excluded for not completing the 7-day food record, leaving the final sample size of the present study to 299 men. General characteristics including anthropometrics, clinical measurements, smoking habits and FA proportions and ratios in serum PL and AT are presented in Table 1. A majority (84%) of men were non-smokers. The median energy intake was 9.3 MJ/d and carbohydrates provided most of the energy intake (44%E), before fats (33%E) and proteins (16%E). The median energy percentage from disaccharides was 11%E of which sucrose was the major contributor (7%E). Alcohol contributed to a median of 4%E (Table 2).

**Table 1:** Anthropometrics, clinical measurements, smoking habits, and fatty acid proportions and ratios in serum and adipose tissue in the study participants

	Median (IQR)^a^
Weight, kg	81.4 (74.7–88.4)
BMI, kg/m^2^	25.4 (23.8–27.7)
Plasma LDL cholesterol, mmol/L	3.6 (2.99–4.32)
Plasma HDL cholesterol, mmol/L	1.58 (1.38–1.84)
Plasma VLDL cholesterol, mmol/L	0.32 (0.17–0.50)
Plasma triglycerides, mmol/L	1.11 (0.80–1.39)
Plasma glucose, mmol/L	4.9 (4.6–5.2)
Systolic blood pressure, mm Hg	135 (123–145)
Diastolic blood pressure, mm Hg	81 (75–86)
Smokers % (no.)	
Nonsmoker	84 (254)
Smoker	16 (47)
Fatty acids in serum PL, (% of total FA)	
Total even-chain SFA^b^	45.8 (45.1–46.3)
16:0	31.7 (31.0–32.6)
16:1	0.64 (0.54–0.75)
SCD, 16:1/16:0	0.020 (0.017–0.023)
Fatty acids in AT (%)	
Total even-chain SFA	29.6 (27.9–31.7)
16:0	22 (21–24)
16:1	7.7 (6.8–9.1)
SCD, 16:1/16:0	0.35 (0.30–0.42)

*BMI* body mass index, *PL* phospholipid, *AT* adipose tissue, *SCD* stearoyl-CoA desaturase

^a^Values are expressed as median (IQR) or  % (*n*)

^b^Total even-chain saturated fatty acids (SFA) equals the sum of 14:0, 16:0 and 18:0

**Table 2:** Macronutrient intake of the study population

	Percentile		
	25	50	75
Total energy, (MJ)	8.0	9.3	10
Total carbohydrate (%E)	40	44	48
Sucrose (%E)	5.7	7.7	10
Disaccharide (%E)	8.5	11	14
Monosaccharide (%E)	4.5	5.6	7.0
Fiber (g/d)	15	19	24
Fiber (%E)	1.7	2.0	2.4
Total fat (%E)	31	33	37
Saturated fat (%E)	13	15	17
Polyunsaturated fat (%E)	3.8	4.3	4.9
Monounsaturated fat (%E)	11	12	14
Protein (%E)	15	16	17
Alcohol (%E)	2.0	4.4	7.9

Intake of energy percentages are presented in 25, 50, and 75 percentiles

After adjusting for BMI, carbohydrate intake was inversely associated with 16:0 in PL (*P* = 0.005) (Table 3). However, associations of carbohydrate-to-fiber ratio with FAs and SCD activity were not evident (BMI-adjusted *P* ≥ 0.13). There was little evidence of linear associations of disaccharide intake with fatty acids or SCD activity (BMI-adjusted *P* ≥ 0.15). However, disaccharide intake was non-linearly associated with 16:1 and SCD activity index in PL (*P* for non-linearity ≤ 0.02), with apparently higher 16:1 and SCD activity at high and low disaccharide intake (Supplementary Fig. 1). In a post hoc exploratory analysis with participants stratified by disaccharide intake (i.e. < 10%E versus ≥ 10%E), disaccharide intake was negatively associated with 16:0 in PL among those with lower intake (*P* = 0.038 after adjustment for BMI), and positively associated with 16:1 and SCD activity in PL among those with higher intakes (*P* = 0.026 and *P* = 0.024, respectively, after adjustment for BMI) (Supplementary Table 1). Monosaccharide intake was not associated with FA or SCD activity after adjusting for BMI. Alcohol consumption was linearly associated with higher levels of 16:0 in PL (BMI-adjusted *P* < 0.001) and with 16:1 and SCD activity (BMI-adjusted *P* < 0.001 and *P* ≤ 0.001, respectively) in both PL and AT (Table 3). In addition, non-linear associations of alcohol intake and 16:1 and SCD activity in PL were evident (*P* for non-linearity ≤ 0.02), with apparently stable levels at low and medium alcohol intake that rapidly increased at higher intakes (Supplementary Fig. 2). Intakes of carbohydrates, disaccharides, monosaccharides, and alcohol were not associated with plasma triglyceride concentrations, neither was the carbohydrate:fiber ratio (Supplementary Table 2).

**Table 3:** Fatty acid proportions and SCD activity in serum phospholipid and adipose tissue per intake tertiles

			Tertile 1	Tertile 2	Tertile 3	*P*_Crude_^d^	*P*_Adjusted_^e^	*P*_Nonlinear_^f^
Carbohydrates	Intake, %E		38.5 (35.8–40.2)	44.4 (43.0–45.9)	49.8 (48.5–52.9)			
	16:0, %^a^	PL	32.0 (31.8–32.3)	31.6 (31.4–31.9)	31.6 (31.4–31.8)	0.006	0.005	0.37
		AT	22.0 (21.7–22.4)	22.5 (22.2–22.9)	22.1 (21.7–22.5)	0.77	0.73	0.13
	Sum^b^ SFA, %	PL	45.9 (45.7–46.1)	45.8 (45.6–46.0)	45.7 (45.6–45.9)	0.26	0.69	0.70
		AT	29.4 (28.9–30.0)	29.9 (29.4–30.5)	29.6 (29.0–30.1)	0.67	0.93	0.31
	16:1, %	PL	0.67 (0.63–0.71)	0.62 (0.59–0.65)	0.64 (0.61–0.67)	0.21	0.37	0.21
		AT	8.24 (7.88–8.59)	7.79 (7.46–8.13)	7.67 (7.33–8.02)	0.02	0.07	0.71
	SCD	PL	0.021 (0.020–0.022)	0.020 (0.019–0.021)	0.020 (0.019–0.021)	0.32	0.53	0.25
		AT	0.38 (0.36–0.40)	0.35 (0.33–0.37)	0.35 (0.33–0.37)	0.06	0.13	0.37
Disaccharides	Intake, %E		7.5 (6.5–8.5)	11.3 (10.5–12.4)	15.6 (14.0–17.0)			
	16:0, %	PL	31.8 (31.6–32.1)	31.6 (31.4–31.8)	31.8 (31.6–32.1)	0.86	0.86	0.06
		AT	22.0 (21.7–22.4)	22.2 (21.8–22.6)	22.4 (22.1–22.8)	0.16	0.16	0.96
	Sum SFA, %	PL	45.8 (45.6–45.9)	45.7 (45.5–45.9)	45.9 (45.7–46.1)	0.26	0.19	0.06
		AT	29.4 (28.8–30.0)	29.5 (28.9–30.1)	30.0 (29.5–30.5)	0.14	0.15	0.69
	16:1, %	PL	0.64 (0.61–0.68)	0.62 (0.60–0.65)	0.66 (0.63–0.69)	0.60	0.57	0.01
		AT	7.97 (7.65–8.29)	7.80 (7.46–8.15)	7.93 (7.55–8.30)	0.87	0.92	0.95
	SCD	PL	0.020 (0.019–0.022)	0.020 (0.019–0.021)	0.021 (0.020–0.022)	0.57	0.56	0.02
		AT	0.37 (0.35–0.39)	0.36 (0.34–0.38)	0.36 (0.34–0.38)	0.57	0.61	0.85
Monosaccharides	Intake, %E		3.8 (3.2–4.5)	5.6 (5.3–6.1)	7.7 (7.0–8.6)			
	16:0, %	PL	31.8 (31.5–32.0)	31.9 (31.6–32.1)	31.6 (31.4–31.8)	0.37	0.36	0.43
		AT	22.1 (21.7–22.4)	22.3 (22.0–22.7)	22.3 (21.9–22.7)	0.38	0.34	0.52
	Sum SFA, %	PL	45.8 (45.6–46.0)	45.8 (45.6–46.0)	45.7 (45.5–45.9)	0.45	1.00	0.82
		AT	29.4 (28.8–29.9)	29.9 (29.3–30.4)	29.6 (29.1–30.2)	0.49	0.76	0.51
	16:1, %	PL	0.66 (0.62–0.70)	0.63 (0.60–0.67)	0.64 (0.61–0.66)	0.35	0.58	0.65
		AT	8.11 (7.72–8.50)	8.01 (7.70–8.33)	7.58 (7.26–7.90)	0.03	0.09	0.81
	SCD	PL	0.021 (0.020–0.022)	0.020 (0.019–0.021)	0.020 (0.019–0.021)	0.36	0.61	0.72
		AT	0.37 (0.35–0.40)	0.36 (0.35–0.38)	0.34 (0.33–0.36)	0.03	0.07	0.84
Carbohydrate:fiber ratio^c^	Intake		10.1 (9.3–10.8)	12.5 (12.1–13.0)	15.5 (14.6–17.4)			
	16:0, %	PL	31.8 (31.6–32.0)	31.8 (31.6–32.0)	31.7 (31.4–31.9)	0.41	0.41	0.15
		AT	22.1 (21.7–22.6)	22.3 (21.9–22.7)	22.2 (21.9–22.6)	0.81	0.83	0.26
	Sum SFA, %	PL	45.7 (45.5–45.9)	45.8 (45.6–46.0)	45.8 (45.6–46.0)	0.36	0.55	0.30
		AT	29.5 (28.9–30.1)	29.8 (29.3–30.4)	29.6 (29.0–30.1)	0.86	0.74	0.18
	16:1, %	PL	0.65 (0.62–0.69)	0.62 (0.59–0.65)	0.65 (0.62–0.69)	0.87	0.99	0.53
		AT	7.90 (7.58–8.22)	7.81 (7.47–8.16)	7.99 (7.62–8.36)	0.69	0.89	0.73
	SCD	PL	0.021 (0.020–0.021)	0.019 (0.019–0.020)	0.021 (0.020–0.022)	0.69	0.83	0.37
		AT	0.36 (0.34–0.38)	0.36 (0.34–0.37)	0.36 (0.35–0.38)	0.86	0.97	0.77
Alcohol	Intake, %E		1.2 (0.2–2.0)	4.4 (3.3–5.2)	10.3 (7.9–14.0)			
	16:0, %	PL	31.4 (31.2–31.6)	31.6 (31.4–31.9)	32.2 (32.0–32.5)	< 0.001	< 0.001	0.53
		AT	22.4 (22.0–22.8)	22.0 (21.6–22.4)	22.3 (21.9–22.6)	0.94	0.90	0.54
	Sum SFA, %	PL	45.7 (45.5–45.8)	45.7 (45.5–45.9)	46.0 (45.8–46.2)	0.01	0.09	0.63
		AT	30.1 (29.5–30.6)	29.4 (28.9–30.0)	29.4 (28.9–29.9)	0.13	0.26	0.55
	16:1, %	PL	0.61 (0.58–0.64)	0.62 (0.59–0.65)	0.70 (0.67–0.74)	< 0.001	< 0.001	0.02
		AT	7.45 (7.10–7.80)	7.76 (7.43–8.08)	8.51 (8.19–8.84)	< 0.001	< 0.001	0.93
	SCD	PL	0.019 (0.019–0.020)	0.019 (0.019–0.020)	0.022 (0.021–0.023)	< 0.001	0.001	0.01
		AT	0.34 (0.32–0.36)	0.36 (0.34–0.38)	0.39 (0.37–0.40)	0.001	0.005	0.58

Intakes are presented as median (IQR) and FA proportions and ratios are presented as mean (95% CI)

*PL* phospholipid, *AT* adipose tissue, *SCD* stearoyl-CoA desaturase, *SFA* saturated fatty acids

^a^Fatty acids proportions are expressed as  % of total FA

^b^Sum of 14:0, 16:0 and 18:0

^c^Carbohydrate:fiber ratio was calculated by dividing carbohydrate intake by fiber intake, both as gram per day

^d^Crude associations were evaluated using a linear regression model with tertile median intake as only independent variables

^e^Associations adjusted for BMI were evaluated using linear regression model with tertile median intake and BMI as independent variables

^f^Nonlinear trends were evaluated using restricted cubic splines with three knots and BMI as a covariate

**Fig. 1:**
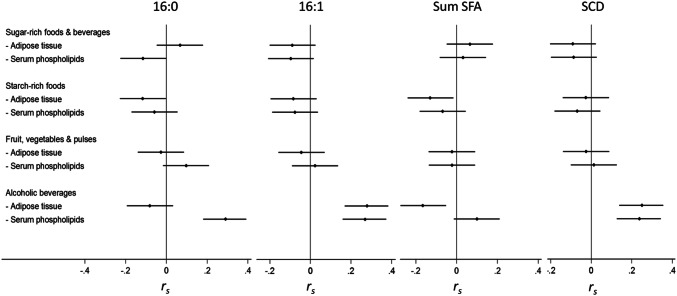
Correlation coefficient and 95% CIs for correlations between fatty acids, sum even-chain SFAs, SCD activity in PL and AT, and food groups

Intake of sugar-rich foods and beverages was weakly and negatively correlated (*r* = − 0.12; *P* = 0.044) with 16:0 in PL but not in AT, and was not correlated with 16:1, total SFA, or SCD activity in PL or AT (Fig. 1). Starch-rich food intake was not correlated with 16:0 (*r* = − 0.06; *P* = 0.34) but correlated negatively with total SFA (*r* = − 0.13; *P* = 0.027) in AT, but not in PL (*r* = 0.07; *P* = 0.24) and was not correlated with 16:1 or SCD activity regardless of FA compartment. Intake of alcoholic beverages correlated with 16:0 in PL (*r* = 0.29; *P* < 0.0001), and with 16:1 (*r* = 0.28, *P* < 0.0001 and *r* = 0.27, *P* < 0.0001 in AT and PL, respectively) and SCD activity (*r* = 0.25, *P* < 0.0001 and *r* = 0.24, *P* = 0.0001 in AT and PL, respectively). Total SFA in AT was negatively correlated with intake of alcoholic beverages (*r* = − 0.17; *P* = 0.004). Intakes of fruits and vegetables were not correlated with FAs and SCD activity (Fig. 1).

Among the 81 men with dietary and gene expression data, no association of dietary carbohydrates, disaccharides, monosaccharides, alcohol, and the carbohydrate-to-fiber ratio with *SCD* gene expression was evident (*P* for linear trend ≥ 0.25; *P* for non-linearity ≥ 0.08) (Table 4).

**Table 4:** Stearoyl-CoA desaturase gene expression in adipose tissue per intake tertiles

	Tertile 1	Tertile 2	Tertile 3	*P*_Crude_^c^	*P*_Adjusted_^c^	*P*_Nonlinear_^c^
Macronutrients						
Carbohydrates	0.23 (0.18–0.31)	0.21 (0.15–0.28)	0.31 (0.22–0.43)	0.23	0.25	0.08
Disaccharides	0.21 (0.15–0.30)	0.30 (0.23–0.40)	0.23 (0.17–0.31)	0.79	0.77	0.94
Monosaccharides	0.26 (0.19–0.35)	0.23 (0.18–0.31)	0.25 (0.17–0.36)	0.96	0.93	0.56
Carbohydrate: fiber ratio^a^	0.25 (0.18–0.35)	0.27 (0.20–0.36)	0.22 (0.17–0.30)	0.72	0.63	0.80
Alcohol	0.23 (0.16–0.32)	0.27 (0.19–0.38)	0.24 (0.19–0.32)	0.56	0.61	0.40
Food groups						
Sugar-rich foods	0.24 (0.19–0.29)	0.26 (0.18–0.37)	0.25 (0.17–0.35)	0.87	0.83	0.73
Starch-rich foods	0.24 (0.18–0.31)	0.26 (0.18–0.37)	0.24 (0.17–0.33)	0.94	1.00	0.83
Fruits and vegetables	0.26 (0.20–0.34)	0.23 (0.17–0.31)	0.25 (0.17–0.37)	0.90	0.86	0.37

Intakes are presented as mean (95% CI)

*SCD* Stearoyl-CoA desaturase

^a^SCD expression is gene expression of SCD

^b^Carbohydrate:fiber ratio was calculated by dividing carbohydrate intake by fiber intake, both as gram per day

^c^*P* value < 0.05 was considered significant for *P* crude, *P* adjusted, and *P* nonlinear; *P* adjusted were evaluated using adjusted model for BMI; *P* Nonlinear, we applied restricted cubic splines with three knots located at 25th, 50th, and 75th percentiles of nutrient intake to explore the association between FAs, SCD activity and nutrient intake

## Discussion

In this Swedish male population, we found no evidence to suggest that higher total carbohydrate or sugar intake is reflected by higher serum or adipose SFA derived from DNL. In fact, there was an inverse association between total carbohydrate intake and 16:0 in serum PL, but not in AT. Alcohol intake was, however, associated with higher 16:0 as well as 16:1 and SCD activity in PL and AT, possibly reflecting an activation of DNL in both liver and AT at higher alcohol intakes [24]. Estimated SCD activity was not associated with carbohydrate intake, and in support to this result there was no significant association between *SCD* gene expression and carbohydrate intake or other macronutrients in this population. To our knowledge, this study is the first to examine the potential associations between carbohydrate intake and circulating and AT fatty acids in a Scandinavian population. Notably, in this population serum and plasma even-chained SFA has been directly linked to incident CVD, diabetes and mortality in other cohorts [12, 13]. Thus, our findings may provide some help to interpret those associations in terms of carbohydrate intake, indirectly suggesting none or only a minor role of high-carbohydrate or sugar-rich foods as drivers of those associations in Swedish populations. The median carbohydrate intake of the current Swedish men was 44% E% which is within the similar range of that reported for the Swedish population at this time [18]. This is, however, lower than the 54E% shown in a US population [25] that reported a positive association between, e.g. 16:0 in plasma PL and increasing intake of total carbohydrates in place of total fat (but not SFA) [10, 25]. Thus, it is possible that the level of carbohydrate intake will determine to what extent circulating SFA may reflect carbohydrate intake in a given population, and thereby also explain the different results between these US and Swedish populations.

In the pan-European EPIC-interact study, it was shown that total even-chain SFA in plasma PL were positively associated with the incidence of type 2 diabetes [11]. There was, however, inconsistent association between the intakes of different types of carbohydrate-rich foods and circulating SFA, thus not clearly supporting that carbohydrates per se were mediating the observed link between PL SFA and diabetes risk [11]. Short-term interventional studies, however, suggest a potential link between certain circulating SFA and high carbohydrate intake. A very low fat diet (10%E) and very high carbohydrate diet was found to markedly up-regulate DNL and increase plasma TG concentrations (with 54% higher proportion of 16:0) compared to a high-fat diet (40%E) [26]. In that study, there was a significant increase in 16:0 when subjects were fed a very high carbohydrate/very low fat diet [26]. Volk et al. instead found that increased carbohydrate intake across a range of intakes promoted an increase in 16:1 in cholesteryl esters and plasma triglycerides, but without consistent dose–response changes in plasma 16:0. Interestingly, 16:0 in plasma PL, in contrast to total SFA including 14:0, seemed to decrease with increasing carbohydrates [27], thus in line with the current observational data that found a carbohydrate intake to be inversely associated with serum 16:0 in PL. It should be noted that in these short-term feeding studies, there were differences in study designs and a clearly different range of total fat and carbohydrate intake that could explain some of the different results. We may speculate that our observational study setting may reflect a more habitual and realistic intake of both fat and carbohydrates, at least in Sweden. However, in line with Volk et al. [27] our post hoc sub-group analyses showed that 6:1n-7 levels in serum PL were associated with a disaccharide intake in the population with higher than 10E % intake. Still, the non-linear relationship between 16:1n-7 and lack of association with the intake of sugar-rich foods warrant careful interpretation of the potential role of 16:1n-7 as a biomarker of excessive sugar intake.

It should be noted that in the present population of Swedish men, the intake of total and simple carbohydrates were fairly representative for a Swedish population at the time [18]. Sucrose was a main source of simple sugars in the present population. Low-fat diets are rarely consumed in Swedish populations, but as such it would be stimulating DNL, especially when mostly consisting of short-chain glucose polymers (75% E), but less so when consisting substantial proportions of starch and complex of carbohydrates [11, 26, 28]. A short-term overfeeding with simple carbohydrates, either fructose or glucose, leads to several potentially deleterious metabolic alterations in healthy human subjects [29]. In overweight subjects, overfeeding with simple carbohydrate markedly increased liver fat and stimulates DNL [30]. In opposite to the present healthy men, sugar intake was not associated with serum 16:0 or total SFA even after adjustment for BMI. Our results accord well with recent data we reported from another cohort (Uppsala Longitudinal Study of Adult Men, ULSAM) of Swedish elderly men [31]. In that study, there was also an inverse association between total carbohydrate and sugar intake, with 16:0 or the SCD product 16:1 in AT [31]. Notably, SFA intake was weakly but significantly directly associated with adipose tissue 16:0 and 16:1, suggesting that in Swedish male populations around the time of the 1990s, higher proportions of SFA and 16:1 in serum and AT do not seem to reflect a higher carbohydrate intake and DNL activity, but more probably a higher dietary SFA and alcohol intake. These relationships, however, need to be studied and replicated in other Swedish populations, especially in younger populations and in women. There are no current data to suggest that the current results would be much different in other European countries, but it is warranted that these associations are also carried out in other countries, as well as in the US and other parts of the world that may have a more excessive consumption of simple sugars than the present Swedish men. Interestingly, when only including individuals (approximately 60% of this population) with higher sugar intake (≥ 10%E  %) there was still no positive association between sugar intake (i.e. disaccharides) and 16:0 levels, although 16:1n-7 and SCD-1 activity index were significantly associated, also after adjustment for BMI. The non-linear relationship between these two latter biomarkers and disaccharide intake, however, warrant further study of both 16:1n-7 and SCD-1 index in PL as potential biomarkers of very high sugar intake.

Our results show an association between higher alcohol intake and higher 16:0, 16:1 and estimated SCD activity. This is in line with previous studies showing that alcohol consumption is positively associated with 16:0 in plasma PL [32] and in erythrocyte membranes [25, 33, 34]. As a potential mechanism, alcohol consumption may increase the activity of acetyl-CoA carboxylase, a key lipogenic enzyme, and thereby the synthesis of 16:0 [33].

We found that total carbohydrate and alcohol intake were associated with lower and higher estimated SCD activity, respectively. However, no significant associations of dietary intake with *SCD* gene expression were observed. Previously, it was shown that SCD activity (in addition to DNL) increased in the short-term during very low fat diets (10%E), while lipogenic gene expression in subcutaneous AT lipogenic was unaffected by such diet [9]. In vivo animal studies suggest that lipogenic genes including SCD and sterol regulatory element-binding protein (SREBP)-1 are up-regulated at high carbohydrate intakes [35]. Thus, our observations in these Swedish men do not accord with such findings.

Our study has several strengths. We used a validated 7-d food record to assess dietary records and the macronutrient composition reported here is representative of the general Swedish population [36], thus increasing generalizability. FA composition was analysed in two biomarker compartments, circulating PL and AT, the latter considered as the preferable compartment to assess long-term dietary habits. All men included in the study were of similar age, thereby reducing the potential bias due to age differences.

Some limitations should be highlighted. The use of a cross-sectional and observational study design does not allow any causal inferences. Although we adjusted the associations for BMI, we cannot rule out residual confounding. Furthermore, we did use a validated 7-d food record, but we acknowledge that possible misreporting may have influenced our findings. Also, we estimated SCD activity in serum and AT, which may not reflect true SCD enzyme activity. In addition, it should be noted that our SCD-1 gene expression analyses was conducted in adipose tissue biopsies from the buttocks, which may not be comparative with those obtained from abdominal adipose tissue. Finally, we did not stratify or adjust for DNL regulatory hormones (e.g. testosterone or insulin levels) due to lack of available data, although it is possible that individual hormonal differences may to some extent influence the associations between carbohydrate intake and fatty acids in the lipogenic pathway.

## Conclusion

Our observations in this population of Swedish men indicated no clear evidence to suggest that high total carbohydrate intake or sugar-rich foods or beverages is reflected by higher DNL-derived SFA in serum PL or AT. Further studies may be needed to investigate if 16:1n-7 in serum PL reflects disaccharides at higher intakes. Instead, alcohol was consistently associated with higher SFA and MUFA. Further population-based studies are needed to confirm these findings, and the effect of quantitative and qualitative carbohydrate intakes on circulating SFA and MUFA should optimally be evaluated in randomized studies in a Swedish population, to allow for casual inference and reduce the impact of inaccurate dietary assessment.

## Electronic supplementary material

Below is the link to the electronic supplementary material.

Supplementary material 1 (DOCX 627 kb)

Supplementary material 2 (DOCX 19 kb)

## References

[1] Risérus U (2008). Fatty acids and insulin sensitivity. Curr Opin Clin Nutr Metab Care.

[2] Hellerstein MK (1996). Regulation of hepatic de novo lipogenesis in humans. Annu Rev Nutr.

[3] Chong M, Hodson L, Bickerton A, Roberts R, Neville M, Karpe F, Frayn K, Fielding B (2008). Parallel activation of de novo lipogenesis and stearoyl-CoA desaturase activity after 3 d of high-carbohydrate feeding. Am J Clin Nutr.

[4] Knopp RH, Retzlaff B, Walden C, Fish B, Buck B, McCann B (2000). One-year effects of increasingly fat-restricted, carbohydrate-enriched diets on lipoprotein levels in free-living subjects. Proc Soc Exp Biol Med.

[5] Raatz SK, Bibus D, Thomas W, Kris-Etherton P (2001). Total fat intake modifies plasma fatty acid composition in humans. J Nutr.

[6] King IB, Lemaitre RN, Kestin M (2006). Effect of a low-fat diet on fatty acid composition in red cells, plasma phospholipids, and cholesterol esters: investigation of a biomarker of total fat intake. Am J Clin Nutr.

[7] Schwarz JM, Neese RA, Turner S, Dare D, Hellerstein MK (1995). Short-term alterations in carbohydrate energy intake in humans. Striking effects on hepatic glucose production, de novo lipogenesis, lipolysis, and whole-body fuel selection. J Clin Invest.

[8] Ma W, Wu JH, Wang Q, Lemaitre RN, Mukamal KJ, Djoussé L, King IB, Song X, Biggs ML, Delaney JA, Kizer JR, Siscovick DS, Mozaffarian D (2015). Prospective association of fatty acids in the de novo lipogenesis pathway with risk of type 2 diabetes: the Cardiovascular Health Study. Am J Clin Nutr.

[9] Chong MF-F, Hodson L, Bickerton AS, Roberts R, Neville M, Karpe F, Frayn KN, Fielding BA (2008). Parallel activation of de novo lipogenesis and stearoyl-CoA desaturase activity after 3 d of high-carbohydrate feeding. Am J Clin Nutr.

[10] Wu JH, Lemaitre RN, Imamura F, King IB, Song X, Spiegelman D, Siscovick DS, Mozaffarian D (2011). Fatty acids in the de novo lipogenesis pathway and risk of coronary heart disease: the Cardiovascular Health Study. Am J Clin Nutr.

[11] Forouhi NG, Koulman A, Sharp SJ, Imamura F, Kroger J, Schulze MB, Crowe FL, Huerta JM, Guevara M, Beulens JW, van Woudenbergh GJ, Wang L, Summerhill K, Griffin JL, Feskens EJ, Amiano P, Boeing H, Clavel-Chapelon F, Dartois L, Fagherazzi G, Franks PW, Gonzalez C, Jakobsen MU, Kaaks R, Key TJ, Khaw KT, Kuhn T, Mattiello A, Nilsson PM, Overvad K, Pala V, Palli D, Quiros JR, Rolandsson O, Roswall N, Sacerdote C, Sanchez MJ, Slimani N, Spijkerman AM, Tjonneland A, Tormo MJ, Tumino R, van der Schouw YT, Langenberg C, Riboli E, Wareham NJ (2014). Differences in the prospective association between individual plasma phospholipid saturated fatty acids and incident type 2 diabetes: the EPIC-InterAct case-cohort study. Lancet Diabetes Endocrinol.

[12] Vessby B, Aro A, Skarfors E, Berglund L, Salminen I, Lithell H (1994). The risk to develop NIDDM is related to the fatty acid composition of the serum cholesterol esters. Diabetes.

[13] Warensjo E, Sundstrom J, Vessby B, Cederholm T, Riserus U (2008). Markers of dietary fat quality and fatty acid desaturation as predictors of total and cardiovascular mortality: a population-based prospective study. Am J Clin Nutr.

[14] Rosell MS, Hellénius M-LB, de Faire UH, Johansson GK (2003). Associations between diet and the metabolic syndrome vary with the validity of dietary intake data. Am J Clin Nutr.

[15] Beynen AC, Katan MB (1985). Rapid sampling and long-term storage of subcutaneous adipose-tissue biopsies for determination of fatty acid composition. Am J Clin Nutr.

[16] Rosell M, Hellénius M-L, Faire Ud, Berglund L, Gustafsson I-B, Johansson G (2003). Contribution of a manually coded part in an optically readable, precoded seven-day food record for the intake of energy, nutrients and foods. Scand J Nutr.

[17] Becker W (1994). Food habits and nutrient intake in Sweden 1989 (in Swedish).

[18] The Swedish National Food Administration (1999). PC-Kost version 1/99.

[19] PC-Kost (1999) Food composition databases. In: Swedish National Food administration, Uppsala, Sweden

[20] Boberg M, Croon LB, Gustafsson IB, Vessby B (1985). Platelet fatty acid composition in relation to fatty acid composition in plasma and to serum lipoprotein lipids in healthy subjects with special reference to the linoleic acid pathway. Clin Sci.

[21] Rosell M, Johansson G, Berglund L, Vessby B, de Faire U, Hellenius ML (2004). Associations between the intake of dairy fat and calcium and abdominal obesity. Int J Obes Relat Metab Disord.

[22] Sjogren P, Sierra-Johnson J, Gertow K, Rosell M, Vessby B, de Faire U, Hamsten A, Hellenius ML, Fisher RM (2008). Fatty acid desaturases in human adipose tissue: relationships between gene expression, desaturation indexes and insulin resistance. Diabetologia.

[23] Gertow K, Rosell M, Sjogren P, Eriksson P, Vessby B, de Faire U, Hamsten A, Hellenius ML, Fisher RM (2006). Fatty acid handling protein expression in adipose tissue, fatty acid composition of adipose tissue and serum, and markers of insulin resistance. Eur J Clin Nutr.

[24] Sozio M, Crabb DW (2008). Alcohol and lipid metabolism. Am J Physiol Endocrinol Metab.

[25] Ma W, Wu JH, Wang Q, Lemaitre RN, Mukamal KJ, Djousse L, King IB, Song X, Biggs ML, Delaney JA, Kizer JR, Siscovick DS, Mozaffarian D (2015). Prospective association of fatty acids in the de novo lipogenesis pathway with risk of type 2 diabetes: the Cardiovascular Health Study. Am J Clin Nutr.

[26] Hudgins LC, Hellerstein M, Seidman C, Neese R, Diakun J, Hirsch J (1996). Human fatty acid synthesis is stimulated by a eucaloric low fat, high carbohydrate diet. J Clin Invest.

[27] Volk BM, Kunces LJ, Freidenreich DJ, Kupchak BR, Saenz C, Artistizabal JC, Fernandez ML, Bruno RS, Maresh CM, Kraemer WJ, Phinney SD, Volek JS (2014). Effects of step-wise increases in dietary carbohydrate on circulating saturated Fatty acids and palmitoleic Acid in adults with metabolic syndrome. PLoS One.

[28] Hudgins LC, Seidman CE, Diakun J, Hirsch J (1998). Human fatty acid synthesis is reduced after the substitution of dietary starch for sugar. Am J Clin Nutr.

[29] Ngo Sock ET, Le KA, Ith M, Kreis R, Boesch C, Tappy L (2010). Effects of a short-term overfeeding with fructose or glucose in healthy young males. Br J Nutr.

[30] Sevastianova K, Santos A, Kotronen A, Hakkarainen A, Makkonen J, Silander K, Peltonen M, Romeo S, Lundbom J, Lundbom N, Olkkonen VM, Gylling H, Fielding BA, Rissanen A, Yki-Järvinen H (2012). Effect of short-term carbohydrate overfeeding and long-term weight loss on liver fat in overweight humans. Am J Clin Nutr.

[31] Iggman D, Arnlov J, Cederholm T, Riserus U (2016). Association of adipose tissue fatty acids with cardiovascular and all-cause mortality in elderly men. JAMA Cardiol.

[32] Saadatian-Elahi M, Slimani N, Chajès V, Jenab M, Goudable J, Biessy C, Ferrari P, Byrnes G, Autier P, Peeters PH, Ocké M, Bueno de Mesquita B, Johansson I, Hallmans G, Manjer J, Wirfält E, González CA, Navarro C, Martinez C, Amiano P, Suárez LR, Ardanaz E, Tjønneland A, Halkjaer J, Overvad K, Jakobsen MU, Berrino F, Pala V, Palli D, Tumino R, Vineis P, Santucci de Magistris M, Spencer EA, Crowe FL, Bingham S, Khaw K-T, Linseisen J, Rohrmann S, Boeing H, Noethlings U, Olsen KS, Skeie G, Lund E, Trichopoulou A, Oustoglou E, Clavel-Chapelon F, Riboli E (2009). Plasma phospholipid fatty acid profiles and their association with food intakes: results from a cross-sectional study within the European Prospective Investigation into Cancer and Nutrition. Am J Clin Nutr.

[33] Fusconi E, Pala V, Riboli E, Vineis P, Sacerdote C, Del Pezzo M, Santucci de Magistris M, Palli D, Masala G, Sieri S, Foggetti CE, Giurdanella MC, Tumino R, Krogh V (2003). Relationship between plasma fatty acid composition and diet over previous years in the Italian centers of the European Prospective Investigation into Cancer and Nutrition (EPIC). Tumori.

[34] Laguzzi F, Riserus U, Marklund M, Vikstrom M, Sjogren P, Gigante B, Alsharari ZD, Hellenius ML, Cederholm T, Frumento P, de Faire U, Leander K (2017). Circulating fatty acids in relation to alcohol consumption: cross-sectional results from a cohort of 60-year-old men and women. Clin Nutr.

[35] Hasty AH, Shimano H, Yahagi N, Amemiya-Kudo M, Perrey S, Yoshikawa T, Osuga J-i, Okazaki H, Tamura Y, Iizuka Y, Shionoiri F, Ohashi K, Harada K, Gotoda T, Nagai R, Ishibashi S, Yamada N (2000). Sterol regulatory element-binding protein-1 is regulated by glucose at the transcriptional level. J Biol Chem.

[36] Becker W, Lennernäs M, Gustafsson I-B, Haraldsdóttir J, Nydahl M, Vessby B, Ytterfors A (1998). Precoded food records compared with weighed food records for measuring dietary habits in a population of Swedish adults. Näringsforskning.

