# Supplementary material for: Association between carbohydrate intake and fatty acids in the de novo lipogenic pathway in serum phospholipids and adipose tissue in a population of Swedish men
Source: Eur J Nutr. 2019 Jul 26;59(5):2089–97. doi: 10.1007/s00394-019-02058-6 (PMC7351873; doi:10.1007/s00394-019-02058-6)
Supplement: Supplementary file 1 — Supplementary material 1 (DOCX 627 kb) [file 394_2019_2058_MOESM1_ESM.docx]

| A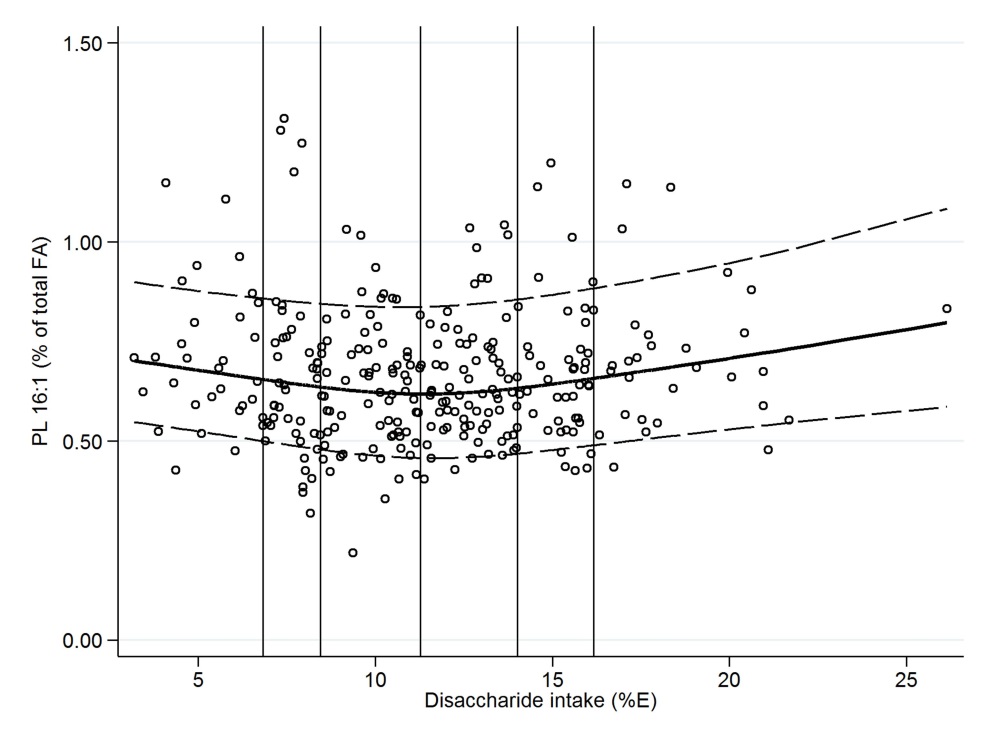A |
| --- |
| B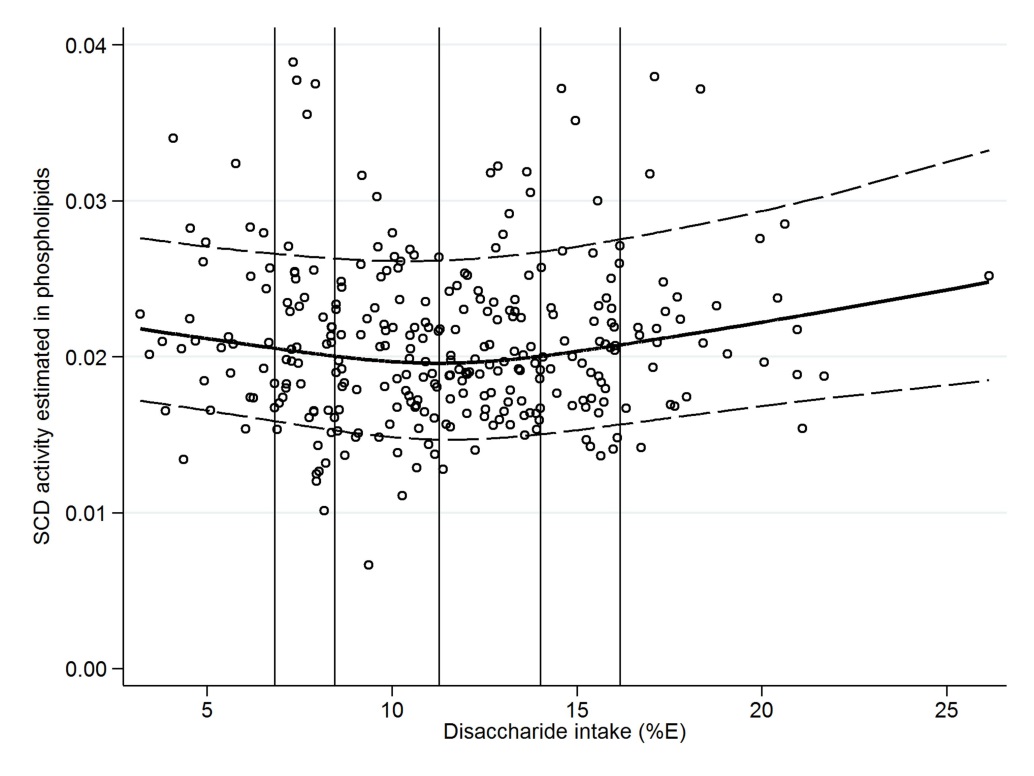 |
| **Figure S1**. Associations of disaccharide intake with 16:1 (A) and estimated SCD activity (B) in serum phospholipids. _____ : regression estimate; ---------: 95% CI; ° : observations; vertical lines: 10, 25, 50, 75, and 90 percentiles. |
| \|  \| \| --- \|   A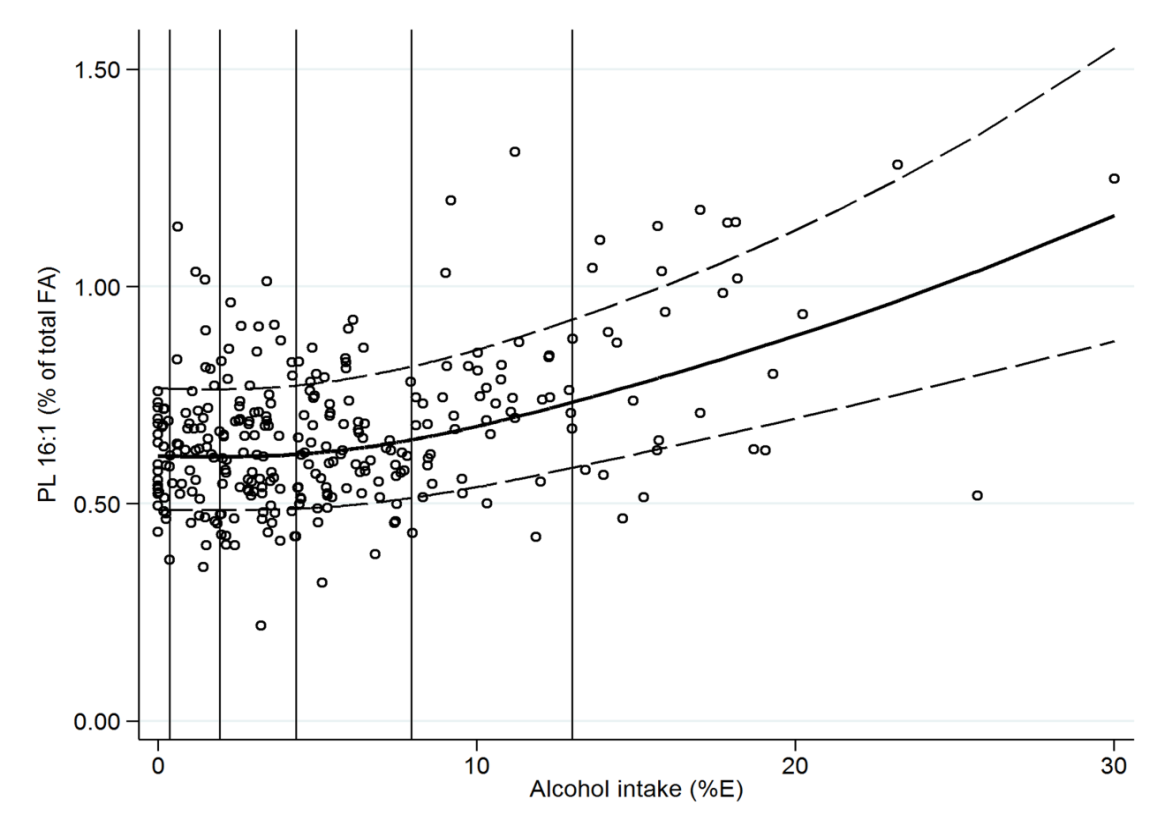 |
| B 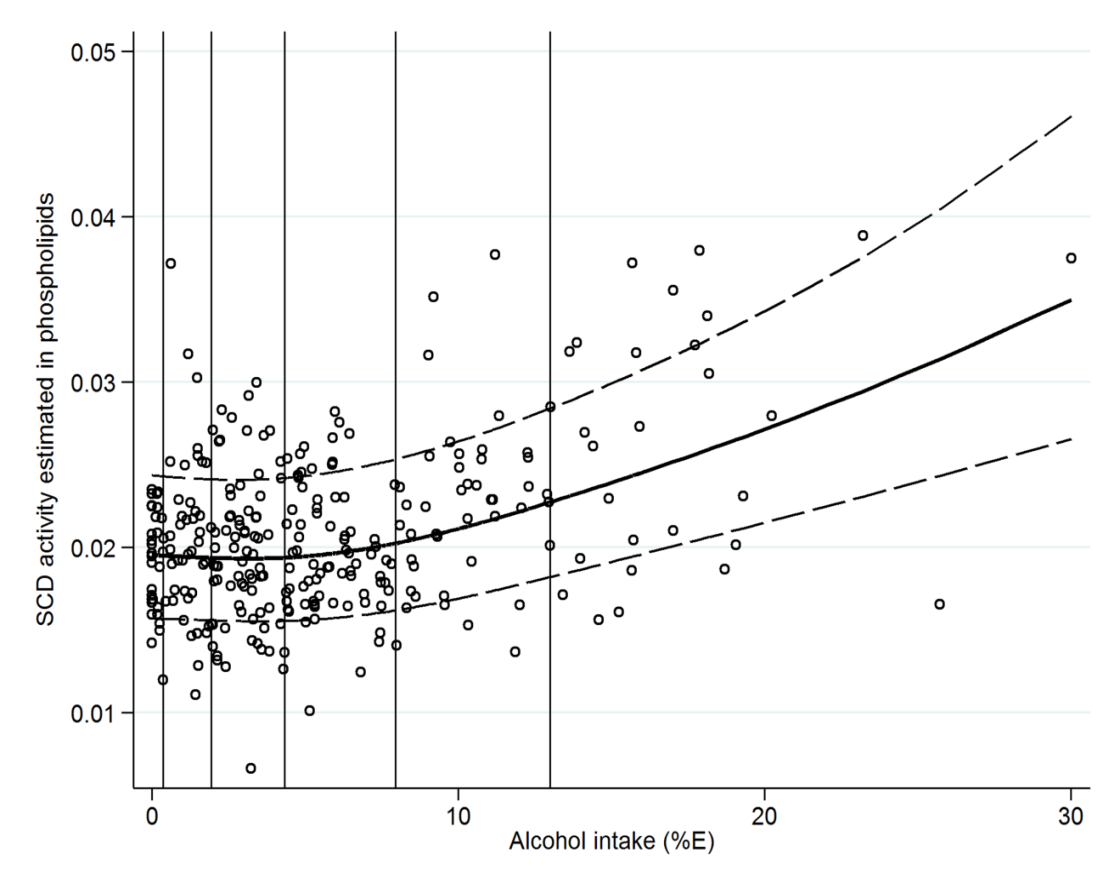 |
| Figure S2. Associations of alcohol intake with 16:1 (A) and estimated SCD activity (B) in serum phospholipids. _____: regression estimate; ---------: 95% CI; ° :observations; vertical lines: 10, 25, 50, 75, and 90 percentiles. |
