# Supplementary material for: Association between carbohydrate intake and fatty acids in the de novo lipogenic pathway in serum phospholipids and adipose tissue in a population of Swedish men
Source: Eur J Nutr. 2019 Jul 26;59(5):2089–97. doi: 10.1007/s00394-019-02058-6 (PMC7351873; doi:10.1007/s00394-019-02058-6)
Supplement: Supplementary file 2 — Supplementary material 2 (DOCX 19 kb) [file 394_2019_2058_MOESM2_ESM.docx]

**Supplementary Table 1.** Associations of disaccharide intake with fatty acids and SCD activity in plasma phospholipids and adipose tissue, stratified by disaccharide intake.^a^

|  |  |  | Disaccharide intake | | | | |
| --- | --- | --- | --- | --- | --- | --- | --- |
|  |  |  | <10%E per day (n=108) | |  | ≥10%E per day (n=183) | |
|  |  | Model | β (95% CI)^b^ | P |  | β (95% CI) | P |
| 16:0, % | PL | Crude^c^ | -0.146 (-0.281; -0.011) | 0.034 |  | 0.015 (-0.045; 0.075) | 0.631 |
|  |  | BMI-adjusted^d^ | -0.145 (-0.281; -0.008) | 0.038 |  | 0.015 (-0.046; 0.075) | 0.634 |
|  | AT | Crude^c^ | 0.067 (-0.158; 0.292) | 0.556 |  | 0.026 (-0.077; 0.128) | 0.623 |
|  |  | BMI-adjusted^d^ | 0.062 (-0.166; 0.291) | 0.589 |  | 0.026 (-0.077; 0.128) | 0.623 |
| Sum SFA, %^e^ | PL | Crude^c^ | -0.037 (-0.151; 0.078) | 0.527 |  | 0.040 (-0.008; 0.088) | 0.098 |
|  |  | BMI-adjusted^d^ | -0.067 (-0.175; 0.041) | 0.219 |  | 0.039 (-0.007; 0.085) | 0.099 |
|  | AT | Crude^c^ | 0.022 (-0.300; 0.345) | 0.891 |  | 0.064 (-0.083; 0.211) | 0.392 |
|  |  | BMI-adjusted^d^ | 0.066 (-0.257; 0.388) | 0.687 |  | 0.062 (-0.084; 0.209) | 0.402 |
| 16:1, % | PL | Crude^c^ | -2.64% (-5.89%; 0.73%)^f^ | 0.122 |  | 1.35% (0.17%; 2.54%)^f^ | 0.025 |
|  |  | BMI-adjusted^d^ | -2.65% (-5.95%; 0.77%)^f^ | 0.126 |  | 1.29% (0.15%; 2.45%)^f^ | 0.026 |
|  | AT | Crude^c^ | -0.037 (-0.223; 0.149) | 0.691 |  | -0.017 (-0.110; 0.077) | 0.724 |
|  |  | BMI-adjusted^d^ | -0.048 (-0.236; 0.140) | 0.613 |  | -0.020 (-0.110; 0.069) | 0.658 |
| SCD | PL | Crude^c^ | -2.13% (-5.27%; 1.13%)^f^ | 0.195 |  | 1.31% (0.18%; 2.44%)^f^ | 0.023 |
|  |  | BMI-adjusted^d^ | -2.16% (-5.35%; 1.14%)^f^ | 0.195 |  | 1.25% (0.17%; 2.35%)^f^ | 0.024 |
|  | AT | Crude^c^ | -0.002 (-0.013; 0.008) | 0.656 |  | -0.001 (-0.006; 0.005) | 0.841 |
|  |  | BMI-adjusted^d^ | -0.003 (-0.014; 0.008) | 0.601 |  | 0.000 (-0.006; 0.005) | 0.860 |
| ^a^16:0, palmitic acid; 16:1, palmitoleic acid; CI, confidence interval; SCD, Stearoyl-CoA desaturase; SFA, saturated fatty acids. 1  ^b^Betas (and 95%CI) indicate the mean change (and 95% CI) in fatty acids levels or SCD activity per 1-%E increase in disaccharide intake.  ^c^Crude associations were evaluated in linear regression models with fatty acids or SCD activity as dependent variables and disaccharide (continuous) as the only independent variable.  ^d^BMI-adjusted associations were evaluated in linear regression models with fatty acids or SCD activity as dependent variables and disaccharide intake and BMI (both continuous) and independent variables.  ^e^Sum of 14:0, 16:0 and 18:0.  ^f^Phospholipid 16:1 and SCD were log-transformed prior to statistical analyses and thus, beta (and 95% CI) represents proportional change per 1-%E increase in disaccharide intake | | | | | | | |
|  | | | | | | | |

**Supplementary Table 2.** Plasma triglycerides by tertile of carbohydrate to fiber ratio and intakes of carbohydrate, disaccharide, monosaccharide, and alcohol.^a^

|  |  | Tertile 1 | Tertile 2 | Tertile 3 | *P*_Crude_^b^ | *P*_Adjusted_^c^ | *P*_Nonlinear_^d^ |
| --- | --- | --- | --- | --- | --- | --- | --- |
| Carbohydrates | Intake, %E | 38.5 (35.8-40.2) | 44.4 (43.0-45.9) | 49.8 (48.5-52.9) |  |  |  |
|  | Plasma triglycerides, mmol/L | 1.09 (0.98; 1.20) | 1.10 (1.01; 1.19) | 1.08 (1.00; 1.17) | 0.954 | 0.411 | 0.967 |
| Disaccharides | Intake, %E | 7.5 (6.5-8.5) | 11.3 (10.5-12.4) | 15.6 (14.0-17.0) |  |  |  |
|  | Plasma triglycerides, mmol/L | 1.07 (0.98; 1.17) | 1.06 (0.97; 1.16) | 1.14 (1.04; 1.24) | 0.323 | 0.248 | 0.680 |
| Monosaccharides | Intake, %E | 3.8 (3.2-4.5) | 5.6 (5.3-6.1) | 7.7 (7.0-8.6) |  |  |  |
|  | Plasma triglycerides, mmol/L | 1.07 (0.98; 1.17) | 1.10 (1.01; 1.20) | 1.10 (1.00; 1.20) | 0.678 | 0.143 | 0.508 |
| Carbohydrate:fiber^e^ | Intake, ratio | 10.1 (9.3-10.8) | 12.5 (12.1-13.0) | 15.5 (14.6-17.4) |  |  |  |
|  | Plasma triglycerides, mmol/L | 1.08 (0.98; 1.19) | 1.05 (0.96; 1.16) | 1.13 (1.04; 1.23) | 0.431 | 0.685 | 0.099 |
| Alcohol | Intake, %E | 1.2 (0.2-2.0) | 4.4 (3.3-5.2) | 10.3 (7.9-14.0) |  |  |  |
|  | Plasma triglycerides, mmol/L | 1.02 (0.95; 1.10) | 1.11 (1.02; 1.22) | 1.14 (1.03; 1.26) | 0.108 | 0.490 | 0.950 |
| ^a^ Intakes are presented as median (IQR) and FA proportions and ratios are presented as mean (95% CI); ^i^ Crude associations were evaluated using a linear regression model with tertile median intake as only independent variables. ^j^ Associations adjusted for BMI were evaluated using linear regression model with tertile median intake and BMI as independent variables. ^l^ Nonlinear trends were evaluated by using restricted cubic splines with 3 knots and BMI as a covariate; ^e^ Carbohydrate: fiber ratio was calculated by dividing carbohydrate intake by fiber intake, both as gram per day. | | | | | | | |
